# Context-Dependency in Relationships Between Herbaceous Plant Leaf Traits and Abiotic Factors

**DOI:** 10.3389/fpls.2022.757077

**Published:** 2022-03-25

**Authors:** Zhenchao Zhang, Jian Sun, Miao Liu, Hua Shang, Jinniu Wang, Jinsong Wang, Huakun Zhou, Yong Li, Yi Wang, Wanjie Chen

**Affiliations:** ^1^Key Laboratory of National Forestry and Grassland Administration on Grassland Resources and Ecology in the Yellow River Delta, College of Grassland Science, Qingdao Agricultural University, Qingdao, China; ^2^State Key Laboratory of Tibetan Plateau Earth System, Resources and Environment, Institute of Tibetan Plateau Research, Chinese Academy of Sciences, Beijing, China; ^3^College of Grassland Science and Technology, China Agricultural University, Beijing, China; ^4^Department of Ecology, Evolution and Natural Resources, Rutgers, The State University of New Jersey, New Brunswick, NJ, United States; ^5^Chengdu Institute of Biology, Chinese Academy of Sciences, Chengdu, China; ^6^Key Laboratory of Ecosystem Network Observation and Modelling, Institute of Geographic Sciences and Natural Resources Research, Chinese Academy of Sciences, Beijing, China; ^7^Key Laboratory of Restoration Ecology for Cold Regions in Qinghai, Northwest Institute of Plateau Biology, Chinese Academy of Sciences, Xining, China; ^8^Beijing Key Laboratory of Wetland Services and Restoration, Institute of Wetland Research, Chinese Academy of Forestry, Beijing, China; ^9^State Key Laboratory of Vegetation and Environmental Change, Institute of Botany, Chinese Academy of Sciences, Beijing, China

**Keywords:** herbaceous plant, leaf trait, growth strategy, abiotic filtering, environmental stress, climatic region

## Abstract

Leaf traits are important indicators of plants’ adaptive strategy to environmental changes. It is an established fact that leaf traits are jointly regulated by climatic and edaphic factors besides genetic factors. However, the relative importance of these abiotic forces in determining the general patterns of herbaceous plant leaf traits across different climatic regions in China is far from clear. We collected 1,653 observations of 542 species of herbaceous plant leaf traits including leaf mass per area, leaf nitrogen, and leaf phosphorus from 316 sampling sites across four climatic regions. We found that the leaf mass per area in the arid region was apparently larger than the others, whereas the smallest mass-based leaf nitrogen and mass-based leaf phosphorus were found in the humid region. Increased growing season temperature and evapotranspiration consistently promoted a conservative growth strategy indicated by higher relative benefit of leaf mass per area, especially in the arid region. Solar radiation in growing season promoted an acquisitive growth strategy indicated by higher relative benefits of mass-based leaf nitrogen and phosphorus in the humid region, but opposite patterns were found in the arid region and semi-humid region. Of all the soil nutrients including soil organic matter, total nitrogen, total phosphorus, and available nitrogen, soil available nitrogen was the strongest predictor of relative benefits of leaf traits associated with a nutrient acquisitive strategy, except in the nutrient-rich semi-humid region. There was a relatively larger number of abiotic factors contributing to relative benefits of leaf traits in the arid and humid regions. We concluded that plant functionality could respond divergently to the same factor facing different habitat conditions. Moreover, the relative benefits of leaf traits tended to be more vulnerable to abiotic filtering in more stressful conditions. Our findings have important implications for understanding the context-dependency of plant functionality to environmental filtering and further improving the predictability of plant dynamics under global change.

## Introduction

Leaf traits are important predictors of evolved plant adaptations to particular inhabiting environments (Violle et al., 2014). They are related to numerous ecological functions: the leaf mass per area (LMA) indicates the investment of photosynthesis and nutrients acquired per unit leaf area; the leaf nitrogen (N) is an essential component of proteins for photosynthesis; and the leaf phosphorus (P) is integral to the nucleic acids and bioenergetics molecules fundamental for metabolism (Wright et al., 2004). Generally, a higher LMA with denser mesophyll tissues and thicker-walled cells allows for a longer leaf lifespan (Wright et al., 2006; Wright and Suttongrier, 2012), whereas higher leaf N and *P*-values, which are associated with higher photosynthetic rates, are observed in leaves with shorter longevity (Gusewell and Koerselman, 2002; Han et al., 2005). The trait-based ecology theory states that environmental changes shape leaf traits (Wang, 2007), which in turn affect ecosystem processes (Van Bodegom et al., 2007; Pearson et al., 2013; Bjorkman et al., 2018), and then soil quality (Buzzard et al., 2019). Variability of leaf traits allows individual plants to adapt to new environmental conditions. The plasticity of traits provides a mechanism for selection by altered environmental conditions that will drive future ecosystem processes.

The adaption and evolution of the leaf traits regulate plant survival and growth under a given set of conditions (Reichstein et al., 2014; Asner et al., 2016). There are two strategies reflecting the trade-off between plant persistence and productivity, and with contrary investment-return patterns, i.e., a slow-growing versus a fast-growing strategy. The former corresponds to greater resource conservation indicated by high relative benefit of LMA, low relative benefit of leaf N and leaf P under a stressful environment (Reich et al., 2003; Carvajal et al., 2019), whereas the latter refers to a quick investment-return pattern that is often associated with rapid resource acquisition indicated by low relative benefit of LMA, high relative benefits of leaf N and leaf P in optimal conditions (Shipley et al., 2006; Wright and Suttongrier, 2012). These strategies provide the most promising methods for determining how an ecosystem functions, such as indicating how the primary productivity and carbon/N cycling vary when facing different environmental conditions. Therefore, obtaining knowledge of leaf traits is a high priority for understanding terrestrial ecosystem adaptations to changing environments, and predicting how ecological processes shift with rapid climate change.

In the past decades, a compelling number of studies have attempted to reveal the relationships between leaf trait and its drivers (Wright et al., 2005; Ordonez and Olff, 2013; Carvajal et al., 2019). Both natural selection (Asner et al., 2016) and genetic constraints (Lusk et al., 2008) control the strength and direction of leaf trait evolution, and the given genetic constraints might play a relatively weaker role in shaping the leaf traits (Reich et al., 2003). In fact, the leaf traits may vary with the plant growth form (Simova et al., 2018; Gong and Gao, 2019), climate (Moles et al., 2014), soil fertility (Asner et al., 2016), taxonomy and phylogeny (He et al., 2009), and sampling scale (Borgy et al., 2017). Thus, no consistent patterns were found from the different studies. For instance, the trait-based ecology theory considers temperature as the central driver of leaf trait variation (Michaletz et al., 2016; Buzzard et al., 2019), but leaf N has been observed to significantly increase (Ordonez et al., 2009; Swenson et al., 2012) or decrease with increasing temperature (Moles et al., 2014; Simova et al., 2017); the soil nutrient supply may play a minor (Bowman et al., 2003) or influential (Ordonez et al., 2009; Reich et al., 2009) role in shaping the leaf traits; and precipitation also shows either strong (Swenson et al., 2012; Asner et al., 2016) or weak (Moles et al., 2014) relationships with leaf traits. To date, there has been no general pattern for the drivers of leaf traits, challenging its universality and predictability (Shipley et al., 2016).

Water and heat availability greatly affect nutrient cycling (Schuur and Matson, 2001), and play crucial roles in mediating plant growth (Sun and Du, 2017; Sun et al., 2020). The favourability hypothesis in biogeography suggests that species in a temperate zone with temperature seasonality should closely follow environmental filtering rules (Fischer, 1960). Previous studies have revealed that species with a narrow geographical range often adapt an extreme drought habitat, with stress-tolerant leaf traits (Thuiller et al., 2004; Geng et al., 2012). Long-term specific water and heat patterns would induce shifts in plant functional compositions (Griffinnolan et al., 2019). That is, the water and heat regime can shape the plant adaptations to the environment, and thereby affect the spatial distribution of the leaf traits (Schwinning and Ehleringer, 2001; Zhang et al., 2017). Though winter climate can affect plant growth especially in more northern latitudes and for cold climate systems, the climatic conditions in growing season play important roles in the growth of plants and thereby their leaf traits.

China has an east-to-west precipitation and temperature gradient as the dominant basis of the climate-region divisions (Zhang and Shen, 2008; Zhang et al., 2016). Meanwhile, herbaceous plants in China cover the broadest geographic range, from a hot arid desert steppe at the lowlands to a cold humid alpine meadow with a ≥4,000 m altitude (Chen et al., 2013), offering an ideal macrocosm for evaluating the spatial pattern of the leaf traits and interpreting plant adaptations to environmental gradients (Westoby and Wright, 2006). However, early studies focusing on the leaf trait response to environmental gradients were mostly performed at small scales (Stubbs and Wilson, 2004; He et al., 2006; Wang, 2007; Werden et al., 2018; Zhang X. L. et al., 2019), and a large-scale leaf trait pattern across China has not been previously reported. We hypothesised that the leaf traits might be divergent and mediated in different ways among different climatic regions across China. In this study, we aimed to address two questions. First, how does China’s herbaceous plant leaf trait change along climatic regions? Second, how do edaphic and climatic factors regulate the leaf traits, specifically in different climatic regions? The answers will advance our knowledge of the ecological filtering and evolution shaping the leaf traits. Moreover, they will help in developing more quantitative and predictive ecological models for incorporating leaf functional traits applicable for China’s terrestrial ecosystems under climate change and anthropogenic activities.

## Materials and Methods

### Data Collection

**FIGURE 1:**
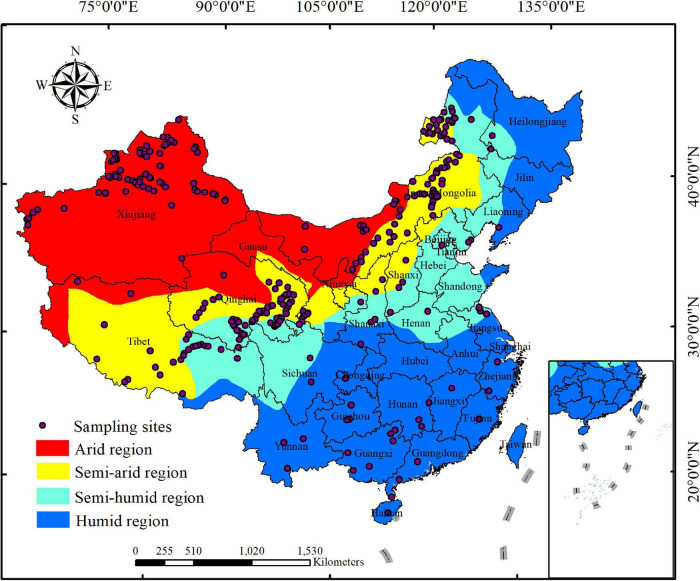
The distributions of sampling sites collected in this study across four China’s climatic regions which are divided based on the method of Thornthwaite (1948) and the data from http://www.resdc.cn/data.aspx?DATAID=253. The inlay included in the current map presents nine-dotted line of China.

The raw data were either obtained from tables or figures (extracted by using the GetData Graph Digitizer, version 2.24)^1^, and a list of all contributing papers were included in Supplementary 2 (Supplementary Material). For each publication, we recorded the following data: site location (longitude and latitude) as well as herbaceous plant LMA, leaf N concentration per mass (N_mass_), and leaf P concentration per mass (P_mass_) as three key leaf functional traits associated with the leaf traits and growing strategies (Diaz et al., 2016). Finally, our database consists of 1,653 observations of 542 species of herbaceous plant from 316 sampling sites across China [shown in Supplementary 3 (Supplementary Material)], with longitude ranging from 74.93°E to 123.92°E, and latitude ranging from 19.10°N to 50.19°N (Figure 1). According to the dry and wet climate-region division in China^2^ (Thornthwaite, 1948), our sampling sites are distributed in four climatic regions: arid, semi-arid, semi-humid, and humid regions in China (Figure 1).

The climate (precipitation, temperature, evapotranspiration, and solar radiation) data were derived from the database of WorldClime 2.0 Beta version 1 (June 2016), as downloaded from http://worldclim.org. They represent average monthly climate data for 1970–2000. The soil attributes [soil organic matter (SOM); total N (STN) and available N (SAN); and total P (STP)] were extracted from a Chinese dataset of soil properties, which was constructed based on a 1:1,000,000 soil map of China and 8,595 soil profiles (Shangguan et al., 2013). Due to the constraint based on data availability and accuracy, there are many measurements of leaf traits at the same soil property level especially for STP. To explore the effects of climatic factors on plant functional traits, we used the mean values from May to September, the growing season, for temperate zone where our sites are mostly located, including the mean growing season precipitation (GSP), mean growing season temperature (GST), mean growing season evapotranspiration (GSE), and mean growing season solar radiation (GSR). Abbreviations of these variables are shown in Table 1. The edaphic and climatic information of the sampling sites were extracted according to geographical location (latitude and longitude) through ArcGIS 10.1 (ESRI, Inc., Redlands, CA, United States). The frequency distributions of the leaf traits (LMA, N_mass_, and P_mass_) were used to ensure the normality of the distribution (Zhou et al., 2019).

**TABLE 1:** Abbreviations of leaf traits, climatic factors, and soil chemical properties.

Variable name	Abbreviation	Unit
Leaf mass per area	LMA	g m^–2^
Leaf nitrogen concentration per mass	N_mass_	mg g^–1^
Leaf phosphorus concentration per mass	P_mass_	mg g^–1^
Growing season temperature	GST	°C
Growing season precipitation	GSP	mm
Growing season evapotranspiration	GSE	mm
Growing season solar radiation	GSR	MJ m^–2^
Soil organic matter	SOM	g kg^–1^
Soil total nitrogen	STN	g kg^–1^
Soil total phosphorus	STP	g kg^–1^
Soil available nitrogen	SAN	mg kg^–1^

### Statistical Analysis

The benefit for a single objective leaf trait is defined as the relative deviation from the mean of a given observation. The relative benefit for the objective leaf trait (LMA, N_mass_, or P_mass_) indicates the relative magnitude of the values of these leaf traits, and is calculated as follows (Bradford and Damato, 2012; Sun et al., 2018):


(1) $$B\,i=\frac{x\,i-x_{\min}}{x_{\max}-x_{\min}}$$

where i is a certain leaf trait (LMA, N_mass_, or P_mass_), *B*_*i*_ is the magnitude of the relative benefit for objective leaf trait *i*, x_i_, x_min_, and x_max_ are the observed value of *i*, minimum, and maximum values for all measured *i*, respectively. A three-dimensional (3D) relative benefit method was used to describe the leaf traits (relative benefits among LMA, N_mass_, or P_mass_) (Li et al., 2019). As shown in Supplementary Figure 1, the vertical distance (l) from the 3D point to the 1:1:1 line represents the magnitude of benefit for the three objective leaf traits, forming 3D coordinates in the LMA-N_mass_-P_mass_ economic spectrum. The relative benefit was decomposed into the LMA (a), N_mass_ (c), and P_mass_ (b) directions, whose values could reflect a plant’s growing strategy (Supplementary Figure 1): a low relative benefit of LMA combined with high relative benefits of N_mass_ and P_mass_ implied a quick investment-return growth strategy; in contrast, a slow investment-return growth strategy was indicated by a high relative benefit of the LMA combined with low relative benefits of N_mass_ and P_mass_.

A principal component analysis (PCA) was performed with all the potential explanatory variables pooled together, to determine the explanatory powers of variance in the leaf functional traits, soil properties, and climatic factors among different climatic regions. The packages of “*FactoMineR*,” “*factoextra*,” and “*corrplot*” in R software (R Core Team, 2016) were used for the PCA. A one-way analysis of variance was performed with SPSS 19.0 software (SPSS Inc., Chicago, IL, United States) to detect differences in the plant, soil, and climate variables among the four climatic regions, using the least significant difference (LSD) as *post hoc* analysis. Regression and correlation analyses were conducted with R Version 3.3.2 (R Core Team, 2016) and SigmaPlot 14.0 software (Systat Software, Inc., Chicago, IL, United States) in each climatic region, to explore the correlations of leaf traits with edaphic and climatic variables. Besides, the correlogram of intercorrelations among relative benefits of leaf traits, soil properties, and climate factors was graphed by “*mgcv*” package in software R (R Core Team, 2016).

## Results

### Variations of Leaf Traits Among the Climatic Regions

**FIGURE 2:**
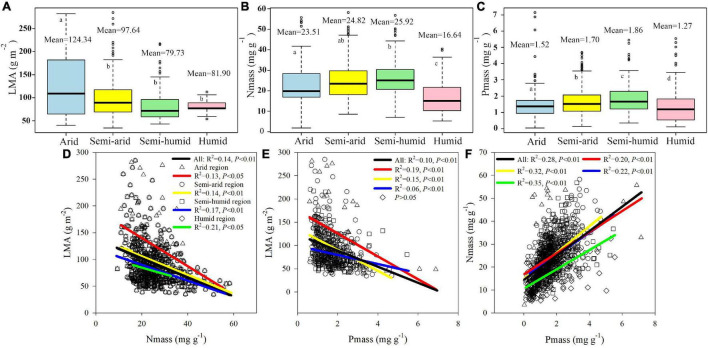
The boxplot **(A–C)** and linear regression relationship **(D–F)** of herbaceous plant LMA, N_mass_, P_mass_ among four China’s climatic regions. The different letters in **(A–C)** represent significance at the 5% confidence level. The black line in **(D–F)** indicates the linear regression of the total data. LMA, N_mass_, and P_mass_ represent leaf mass per area, mass-based leaf nitrogen, and mass-based leaf phosphorus, respectively.

The LMA in the arid region, with a mean value of 124.34 g m^–2^, was significantly higher than the mean values of 97.64, 81.90, and 79.73 g m^–2^ for the semi-arid, semi-humid, and humid regions, respectively (*P* < 0.05, Figure 2A). N_mass_ and P_mass_ tended to initially increase and then decrease from arid to humid regions. The average values of N_mass_ were 23.51, 24.82, 25.92, and 16.64 mg g^–1^ for the arid, semi-arid, semi-humid, and humid regions, respectively (Figure 2B). P_mass_ was significantly different among the four climatic regions (*P* < 0.05), with mean values of 1.52, 1.70, 1.86, and 1.27 mg g^–1^ for the arid, semi-arid, semi-humid, and humid regions, respectively (Figure 2C). The LMA was negatively associated with N_mass_ and P_mass_, with the deepest slope in the arid region (Figures 2D,E), whereas N_mass_ was positively correlated with P_mass_ (Figure 2F).

### Variations of Edaphic and Climatic Factors in Different Climatic Regions

The PCA results indicated that two components explained 57.4% of the total variance (Supplementary Figure 2). Specifically, the first principal component (PC1) was highly relevant to the GSP, GSE, GSR, N_mass_, and P_mass_; the second principal component (PC2) was highly relevant to the SOM, STN, SAN, SAP, and LMA (Supplementary Figure 2). For the soil properties, SOM, STN, STP, and SAN presented a unimodal pattern along climatic regions, and peaked in the semi-humid region, with average values of 4.77, 0.23, 0.08, and 173.65 mg kg^–1^, respectively (Supplementary Table 1). For the climatic variables, from arid to humid region, the GST initially decreased, and then increased with a minimum value of 9.55°C in the semi-humid region, while there was a significant decrease in the GSR (Supplementary Table 1).

### Effects of Edaphic and Climatic Variables on Leaf Traits in Different Climatic Regions

**FIGURE 3:**
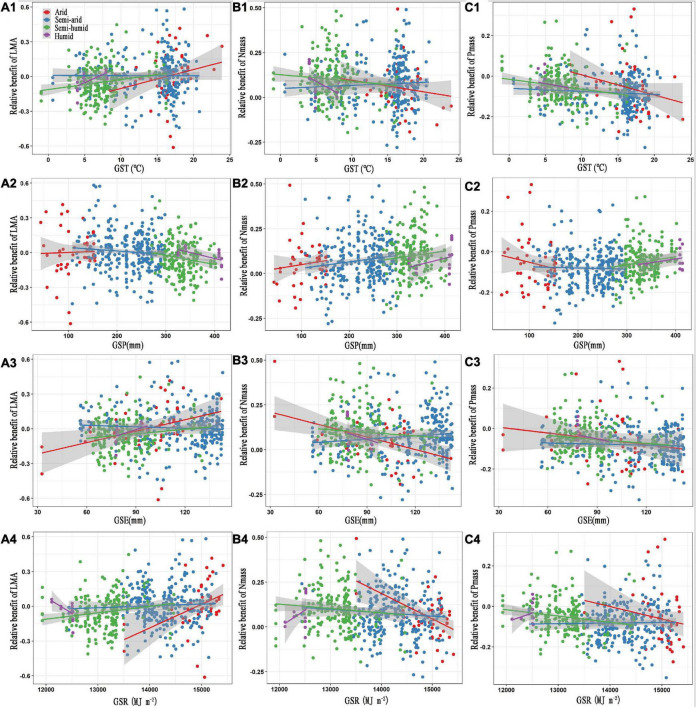
Relationships between the relative benefits of leaf traits with growing season temperature **(A1–C1)**, precipitation **(A2–C2)**, evapotranspiration **(A3–C3)** and solar radiation **(A4–C4)**. The red, blue, green, and purple colours represent the arid, semi-arid, semi-humid, and humid climatic regions, respectively. LMA, leaf mass per area; N_mass_, mass-based leaf nitrogen; P_mass_, mass-based leaf phosphorus; GST, growing season temperature; GSP, growing season precipitation; GSE, growing season evapotranspiration; GSR, growing season solar radiation. The statistical information of linear regression are shown in Table 2.

In regard to the climatic factors, GST, GSE, and GSR positively affected the relative benefit of the LMA, but negatively influenced the relative benefits of N_mass_ and P_mass_ in the arid and semi-humid regions, similar to the effect of GST in the humid region (*P* < 0.05, Figures 3A1–C1, A3–C3, A4–C4 and Table 2). However, the relationship patterns of the GSR with the leaf traits in the humid region were converse to those in the arid and semi-arid regions (Figures 3A4–C4). The GSP showed weak negative correlations with the relative benefits of the LMA but was positively associated with the relative benefits of N_mass_ and P_mass_ in the semi-arid and semi-humid regions (Figures 3A2–C2).

**TABLE 2:** Statistical information of linear regression relationships between the relative benefits of leaf traits and climatic factors.

Climatic variables	Climatic region	Relative benefit of LMA				Relative benefit of N_mass_				Relative benefit of P_mass_			
		*R*	Slope	Intercept	*P*	*R*	Slope	Intercept	*P*	*R*	Slope	Intercept	*P*
Growing season temperature	Arid	0.25	0.02	–0.27	*	0.18	–0.01	0.16	*	0.24	–0.01	0.11	*
	Semi-arid				0.93				0.30				0.19
	Semi-humid	0.19	0.009	–0.11	**				0.15	0.19	–0.005	–0.02	**
	Humid	0.63	0.03	–0.19	*	0.45	–0.02	0.20	*				0.46
Growing season precipitation	Arid				0.87				0.41				0.32
	Semi-arid	0.09	–0.0003	0.08	*	0.14	0.0004	–0.007	*				0.60
	Semi-humid	0.17	–0.0008	0.21	*				0.16	0.15	0.0004	–0.18	*
	Humid				0.08				0.31				0.35
Growing season evapotransp-iration	Arid	0.36	0.003	–0.32	*	0.46	–0.002	0.28	**	0.17	–0.001	0.04	*
	Semi-arid				0.28				0.20				0.38
	Semi-humid	0.16	0.001	–0.16	*				0.16	0.14	–0.0006	0.004	*
	Humid				0.09				0.35				0.35
Growing season solar radiation	Arid	0.39	0.0002	–3.04	***	0.48	–0.0001	2.18	***				0.22
	Semi-arid				0.42				0.20				0.77
	Semi-humid	0.19	0.00006	–0.77	**				0.15	0.18	–0.00003	0.35	**
	Humid	0.57	–0.0003	3.39	*				0.23				0.36

*P < 0.05, **P < 0. 01, ***P < 0.001.

**FIGURE 4:**
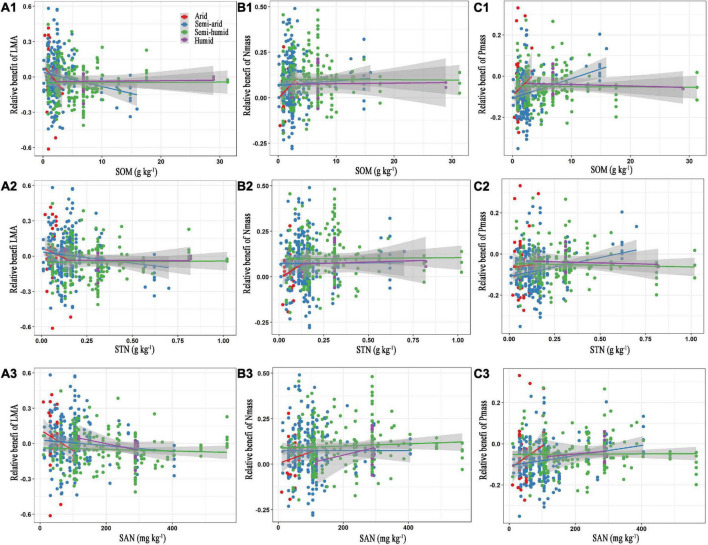
Relationships between the relative benefits of leaf traits with soil organic matter **(A1–C1)**, total nitrogen **(A2–C2)** and available nitrogen **(A3–C3)**. The red, blue, green, and purple colours represent the arid, semi-arid, semi-humid, and humid climatic region, respectively. LMA, leaf mass per area; N_mass_, mass-based leaf nitrogen; P_mass_, mass-based leaf phosphorus; SOM, soil organic matter; STN, soil total nitrogen; SAN, soil available nitrogen. The statistical information of linear regression are shown in Table 3.

**FIGURE 5:**
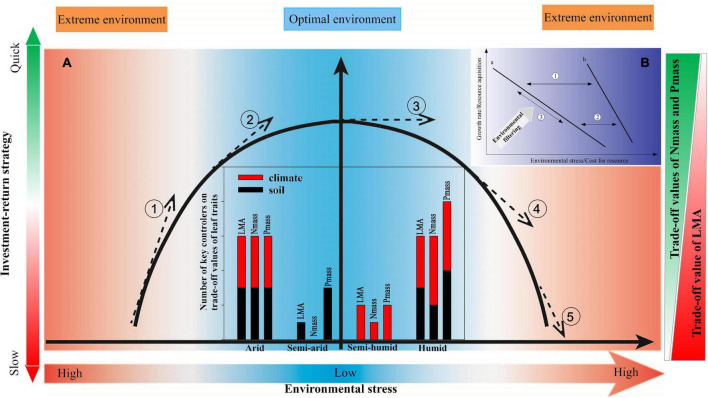
Conceptual frameworks of relationship between leaf traits and environment reflect changes in the responses of plant growing strategies to environmental conditions. Subgraph **(A)** the relative benefit of LMA decreases while relative benefits of N_mass_ and P_mass_ increase when the environmental stress weakens, indicating an investment-return strategy changing from slow to quick (① and ②), ④ and ⑤ represent opposite trends from optimal to extreme environmental conditions; the leaf traits are more sensitive to environmental drivers with steeper slopes (① and ⑤) in an extreme environment than in an optimal environment (②, ③, and ④). The bar chart shows the numbers of the key climatic and edaphic factors controlling the relative benefits of leaf traits, with R square values no less than 0.20 according to the correlogram of intercorrelations (Supplementary Figure 5); the red and black colours represent climatic and edaphic factors, respectively. LMA, leaf mass per area; N_mass_, mass-based leaf nitrogen; P_mass_, mass-based leaf phosphorus. The shades of the colours represent the investment-return rate, the extent of environmental stress, and the magnitude of relative benefits of LMA, N_mass_, and P_mass_, respectively. Subgraph **(B)** a conceptual framework of environmental stress and cost for resource-regulating plant growth rate and resource acquisition indicated by leaf traits. Arrow ① and ②: the relationship curve turns but keeps a downward sloping trend when the environmental stress and costs for resources shift. The slope of curve b under high environmental stress is generally steeper than that of curve a in a less stressful environment. Arrow ③: plant growth rate and resource acquisition slide along curve a or b driven by environmental filtering. For example, as the environmental stress characterised by low resource availability strengthens, the cost for resources increases, so that the plants select a conservative strategy with a slow growth rate and long-life span.

For the edaphic factors, SOM, STN, and STP were negatively related to the relative benefit of the LMA, but positively affected the relative benefits of N_mass_ and P_mass_ in the semi-arid regions (Figures 4A1–C2 and Supplementary Figure 7). Similar correlations were observed between the SAN with the relative benefits of the LMA, N_mass_, and P_mass_, except in the semi-humid region (Figures 4A3–C3). The number of key edaphic and climatic factors driving the leaf traits in the arid and humid regions was greater than those in the semi-arid and semi-humid regions (Figure 5).

**TABLE 3:** Statistical information of linear regression relationships between the relative benefits of leaf traits and edaphic factors.

Edaphic variables	Climatic region	Relative benefit of LMA				Relative benefit of N_mass_				Relative benefit of P_mass_			
		*R*	Slope	Intercept	*P*	*R*	Slope	Intercept	*P*	*R*	Slope	Intercept	*P*
Soil organic matter	Arid				0.16				0.11				0.33
	Semi-arid	0.19	–0.01	0.039	**				0.38	0.29	0.01	–0.11	***
	Semi-humid				0.90				0.94				0.92
	Humid				0.88				0.91				0.67
Soil total nitrogen	Arid				0.33				0.08				0.82
	Semi-arid	0.13	–0.19	0.04	*				0.95	0.24	0.18	–0.11	***
	Semi-humid				0.84				0.88				0.57
	Humid				0.95				0.81				0.76
Soil total phosphorus	Arid				0.44				0.11				0.96
	Semi-arid	0.16	–1.42	0.10	**	0.12	81	0.02	*	0.14	0.61	–0.12	*
	Semi-humid				0.38				0.50				0.59
	Humid				0.97				0.82				0.74
Soil available nitrogen	Arid	0.26	–0.002	0.12	*	0.2	0.0006	0.004	*	0.25	0.001	–0.12	*
	Semi-arid	0.10	–0.0002	0.03	*				0.99	0.20	0.0002	–0.11	***
	Semi-humid				0.36				0.35				0.84
	Humid	0.48	–0.0006	0.11	*				0.27				0.52

*P < 0.05, **P < 0. 01, ***P < 0.001.

## Discussion

### Variations of Leaf Traits in Different Intervals Across Climatic Regions

Our study showed that the LMA in the arid region was drastically higher than those in other regions (Figure 2A), indicating that plants in hyper-arid conditions invest more photosynthate per unit leaf area. This result is in agreement with many previous studies indicating that plants inhabiting arid areas have a high LMA as manifested by thicker leaves with denser mesophyll (Fonseca et al., 2000; Niinemets, 2001; Wright et al., 2001), often combined with smaller and thicker-walled cells for reducing water loss (Wright et al., 2006). As the maximum photosynthetic rate of plants in a dry environment is relatively lower at the same level of leaf N content, the high LMA increases the photosynthetic tissue per unit area supporting photosynthesis, and allows for continued leaf function in arid conditions (Evans and Poorter, 2001). In addition, the SOM, STN, STP, and SAN in the arid region are significantly lower than those in other regions (Supplementary Table 1), and the low available water decreases the soil nutrient availability (Sardans and Penuelas, 2004) and further restricts plant nutrient absorption (Farooq et al., 2009). Consequently, survival is the primary goal for plants, which tend to select a relatively conservative investment strategy. The higher LMA in the arid region should be interpreted as an adaptive strategy of plants against extreme environmental stress, which was alleviated in other climatic regions, and the LMA was relatively smaller.

The leaf N and P values play pivotal roles in photosynthesis and protein synthesis, respectively (Niklas et al., 2005; Westoby and Wright, 2006). The N_mass_ and P_mass_ values in the humid region were dramatically lower than those in the other regions (Figures 2B,C), indicating a small carbon gain rate. This is probably because frequent strong precipitations accelerated the soil erosion and nutrient losses in the humid region, indicated by that the soil nutrients were significantly lower than those in the semi-humid region (Supplementary Table 1). Moreover, the soils were often waterlogged and anoxic, depressing the oxidative metabolic processes of the roots and microbes in the mesic lands, where the plant growth rates were greatly inhibited by excessive rainfall (Zhang S. S. et al., 2019; Zheng Q. et al., 2019).

We observed significantly negative correlations of the LMA with N_mass_ and P_mass_ (Figures 2D,E), which agrees with previous studies demonstrating that a higher LMA is often associated with lower leaf N and P concentrations, indicating slow growth rates (Santiago and Wright, 2007; Donovan et al., 2011). Nevertheless, the leaves with a high LMA and low N_mass_ and P_mass_ are more tolerant to unfavourable growing conditions. In contrast, plants would be vulnerable to damages from environmental stress if a high LMA was combined with high N_mass_ and P_mass_, as it would accelerate energy losses *via* respiration (Wright et al., 2004). This might explain why negative-relationship slopes were the deepest in the arid region (Figures 2D,E).

### Edaphic and Climatic Variables Regulating Leaf Traits

The favourability hypothesis suggests that plants tend to choose certain optimum trait assemblages when facing particular inhabiting conditions, either through trait plasticity within a species or via species turnover in community composition (Swenson et al., 2012; Simova et al., 2015). Temperature which influences plant hydraulics, nutrient utilization, and leaf energy balance, is a central controller of leaf trait variation (Fonseca et al., 2000; Michaletz et al., 2016). In the present study, it was obvious that the orientations of GST and LMA exhibited the smallest angle (Supplementary Figure 2), indicating that the GST tended to promote an increase in the LMA, which conflicts with prior studies (Swenson et al., 2012; Simova et al., 2015; Simova et al., 2018). We propose two probable explanations for the contradictory results. First, the sampling sites in our study covered a relatively narrower GST range (from –1 to 28.63°C) than the former studies. The plant growth form was mainly herbaceous plant species in the temperate zone, unlike, e.g., boreal coniferous species, with higher LMAs in cold conditions for modulating the temperatures of leaves (Michaletz et al., 2016). Second, the soil nutrients such as SOM, STN, STP, and SAN apparently decreased with increases in GST (Supplementary Figure 4). Plants exhibited low potential for resource capture and a slow photosynthetic rate under the more unfavourable inhabiting conditions (Wright et al., 2010; Swenson et al., 2012). They can also explain why the GST negatively influences P_mass_ and thereby affects the photosynthesis-N relationship (Reich et al., 2009), in accordance with previous studies (Kerkhoff et al., 2005; Sun et al., 2019). In addition, the negative correlation of the GST with N_mass_ (Supplementary Figure 2) provides evidence for the temperature–plant physiological hypothesis, i.e., leaf N would increase with decreasing temperature, as a high N concentration could compensate for physiological inefficiency in a cold environment (Reich and Oleksyn, 2004).

In this study, we used the benefit metrics for leaf traits to determine changes in plant growth strategy by analysing their relationship with different environmental drivers. Specifically, low relative benefits of LMA, high relative benefits of N_mass_ and P_mass_ implied an acquisitive growth strategy; conversely, a conservative growth strategy. Consistent with previous studies indicating that the responses of plant physiological processes to the same factor and underlying rules may be largely divergent in face of different environments (Quan et al., 2019), we found that the relative benefit of LMA was distinctly elevated, while the relative benefits of N_mass_ and P_mass_ decreased with increasing GSR in the arid and semi-humid regions, whereas in the humid region, the leaf traits showed the opposite pattern (Figures 3A4–C4). In that regard, the GSR is strongly positively associated with the GST and GSE in the arid and semi-humid regions and displays a significantly negative relation to the GSP in the semi-humid region (Supplementary Figures 3D–F). The radiation-induced warming leads to the reduction of soil water through intensifying evaporation (Wang et al., 2009), thereby aggravating the negative influences of drought in the arid area (Quan et al., 2019). Moreover, an increase in the GSE with increasing GSR accelerates the water consumption from transpiration (Decker et al., 2013). As the GSR increases, plants are expected to adopt conservative investment-return strategies with slow growth rates and higher LMAs with smaller areas exposed to solar radiation per gram leaf, to reduce radiative heating and water loss (Wright et al., 2004; Chen et al., 2013; Moles et al., 2014).

However, for the humid region, the overall relationships of the GIR with the GST, GSP, and GSR are considerably weaker than those in the other regions (Supplementary Figure 3). Indeed, the GIR is the lowest in this region (Supplementary Table 1), implying a relatively limited light resource availability. As a vital factor directly influencing plant growth, solar radiation plays crucial roles in many plant physiological processes, such as leaf N and P accumulations (Takashima et al., 2004; Sun et al., 2019), photosynthesis (Waite and Sack, 2010), and protein synthesis (Xu, 2003). Consistent with Wright et al. (2006), who revealed that plants in higher-irradiance regions had a lower LMA and higher photosynthetic capacity than those in lower-irradiance areas, we observed that the GSR presented a negative impact on the relative benefit of LMA, but remarkably positively affected the relative benefits of N_mass_ and P_mass_ in the radiation-lacking region. This indicated that the plant growing strategy shifted from slow to fast as the GSR increased.

Unexpectedly, the GSP showed no significant effect on the relative benefit of leaf traits in the arid region (Figures 3A2–C2). The following several mechanisms might contribute to this. First, although precipitation data are relatively easier to obtain, they are not always sufficient proxies for soil water availability (Hickler et al., 2009), especially in hyper-arid grassland mostly belonging to desert steppe, where the soil water holding capacity are small. Thus, the soil available water is still limited, even if there is relatively high rainfall (Li et al., 2008; Xie, 2011). Second, it may be attributed to the inherent characteristics of plant species evolutionarily adapted to the hyper-arid conditions, with corresponding trait syndromes such as a high LMA and small stature (Wellstein et al., 2017), thereby inducing the plant community to be relatively less sensitive to rainfall (White et al., 2000). Finally, the low soil nutrient contents in this region (Supplementary Table 1) greatly restrict plant growth, which might weaken the relative importance of the GSP in mediating the leaf traits.

Soil nutrients play positive roles in multiple plant physiological activities. For example, the SOM provides energy for heterotrophic N-fixing microbes, and further facilitates plant N fixation (Reed et al., 2011; Zheng M. H. et al., 2019); the soil N can strongly affect the leaf N and plant photosynthesis (Lu et al., 2011; Yue et al., 2019); and a low soil P may lead to a lower leaf P and thus limit plant function (Han et al., 2005; Quesada et al., 2009; Sun et al., 2019). Plants invest more energy for resource acquisition in nutrient-insufficient soil (Nasto et al., 2014). Soil nutrient changes can induce species turnover, by influencing plant facilitation and competitive exclusion (Marriott et al., 2002; Guo, 2003). The regression relation between relative benefits of each leaf trait and soil nutrients indicated that the SOM, STN, and STP consistently presented significant promotion of an acquisitive growth strategy in the semi-arid region (Figure 4, Table 3, and Supplementary Figure 7). This may be ascribed to the poorer soil quality in this region (Supplementary Table 1). In contrast, the soils in the semi-humid region were relatively more fertile, providing plentiful nutrients for plant growth. In that regard, the leaf traits will not respond to the nutrient supply after the soil fertility fully meets the plant demand (Figure 5③; Bowman et al., 2003).

We found no evidence that the SOM, STN, and STP caused variations of relative benefits of leaf traits in the arid region with insufficient soil nutrients (Figure 4 and Supplementary Figure 7). This may be owing to the narrow ranges of the SOM, STN, and STP content. Moreover, drought constrains the effectiveness of soil fertility on plant growth, and thus the sensitivity of the leaf traits to nutrients in the hyper-arid area (Sardans and Penuelas, 2004; Farooq et al., 2009). Additionally, the SAN presented negative effects on the relative benefit of LMA, but positively affected the relative benefits of N_mass_ and P_mass_, promoting an acquisitive investment strategy except in the SAN-rich semi-humid region (Figures 4A3–C3). This agrees with early studies, i.e., that the leaf traits are more related to the N supply at a global scale (Ordonez et al., 2009).

Overall, our results confirm that abiotic filtering shapes the leaf trait pattern, i.e., higher costs for resources and environmental stress lead to a slow-growing strategy with conservative leaf traits, whereas optimal conditions result in a fast tissue turnover with acquisitive leaf traits across climatic regions in China (Figure 5). This supports the favourability hypothesis, i.e., that the plant leaf traits should be governed mainly via environmental filtering in a climatically harsh temperate zone (Fischer, 1960). Furthermore, more key environmental drivers and steeper leaf-trait-environment relationship slopes were found in the arid and humid regions (Figure 5), suggesting that the degree of abiotic filtering is stronger in more stressful environments characterised by e.g., nutrient limitations or drought or over-wet conditions, in line with previous studies (Swenson et al., 2012; Simova et al., 2015). In that regard, the more stressful ecosystems are commonly inhabited by stress-tolerant species with a narrow geographical range size owing to natural selection (Thuiller et al., 2004), which respond more sensitively to environmental changes than wide-ranging species (Geng et al., 2012). Together with the finding that the environmental signal for the leaf traits would be relatively weaker in the optimal environmental conditions represented by the semi-humid region (Supplementary Figure 6), we conclude that an extreme environment elicits extreme ecological responses (Zhang F. Y. et al., 2019) and predict that frequent extreme climatic events caused by future climate change may enhance the fluctuations of the leaf traits in the terrestrial ecosystems (Jentsch and Beierkuhnlein, 2008; Stott et al., 2016).

## Conclusion

Our findings provide valuable information for the emerging field of functional biogeography, particularly across the different climatic regions in China. First, the large LMA and small N_mass_ and P_mass_ were observed in the arid region, implying that plants under environmental stress as characterised by drought and low nutrient availability are slow-growing, with a conservative investment-return strategy. In contrast, acquisitive leaf traits were found in the semi-humid region, which presented optimal inhabiting conditions. Second, the GST and GSE consistently promoted conservative leaf traits, especially in the arid region. However, the GSR induced an opposite leaf trait pattern between the humid region and the others, confirming that plant functionality could respond divergently to the same factor in the face of different habitat conditions. Of all the studied soil nutrients, the SAN was the best indicator of a large-scale leaf trait pattern. Finally, we revealed that the leaf-trait-environment relationships were stronger under more stressful environmental conditions. These findings will help in refining ecological models and improving their applicability for China’s ecosystems, further enabling us to evaluate the influence of global change. Owing to limited data availability, we only focused on the spatial patterns of the leaf traits and environmental controls with all herbaceous species pooled together; the influences of biotic interactions were not examined in the current study. Further in-depth systematic studies to evaluate the co-effects of abiotic and biotic factors on the leaf traits while considering the plant growth form are therefore highly desirable.

## Data Availability Statement

The raw data supporting the conclusions of this article will be made available by the authors, without undue reservation.

## Author Contributions

ZZ and JS conceived the study and wrote the manuscript. ZZ, JS, ML, HS, JNW, and JSW collected and analysed the data. ZZ, JS, ML, JNW, HZ, and YW drew the graphs. All authors reviewed and revised the manuscript.

## Conflict of Interest

The authors declare that the research was conducted in the absence of any commercial or financial relationships that could be construed as a potential conflict of interest.

## Publisher’s Note

All claims expressed in this article are solely those of the authors and do not necessarily represent those of their affiliated organizations, or those of the publisher, the editors and the reviewers. Any product that may be evaluated in this article, or claim that may be made by its manufacturer, is not guaranteed or endorsed by the publisher.

## Footnotes

1
http://www.getdata-graph-digitizer.com/

2
http://www.resdc.cn/data.aspx?DATAID=125
