# Supplementary material for: Context-Dependency in Relationships Between Herbaceous Plant Leaf Traits and Abiotic Factors
Source: Front Plant Sci. 2022 Mar 25;13:757077. doi: 10.3389/fpls.2022.757077 (PMC8990845; doi:10.3389/fpls.2022.757077)
Supplement: Supplementary file 1 [file Data_Sheet_1.docx]

Supplementary Material 1

# Supplementary Figures and Tables

## Supplementary Figures


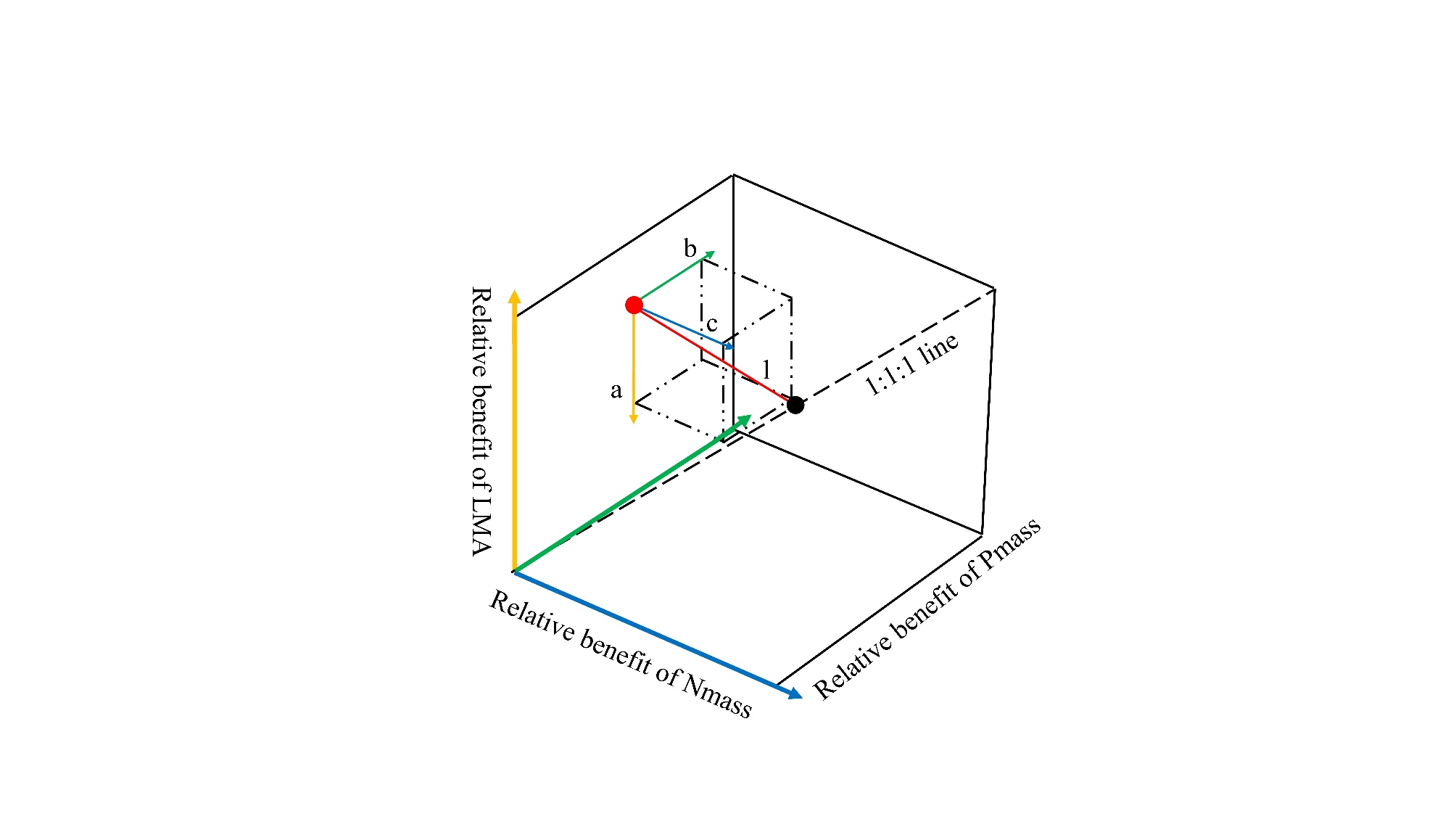


**Supplementary Figure 1.** Illustration and example of relative benefits among three objective leaf traits (LMA, N_mass_, and P_mass_). The 1:1:1 line is described as the line in which the benefits of three objects are equal. LMA: leaf mass per area; N_mass_: mass-based leaf nitrogen; P_mass_: mass-based leaf phosphorus.


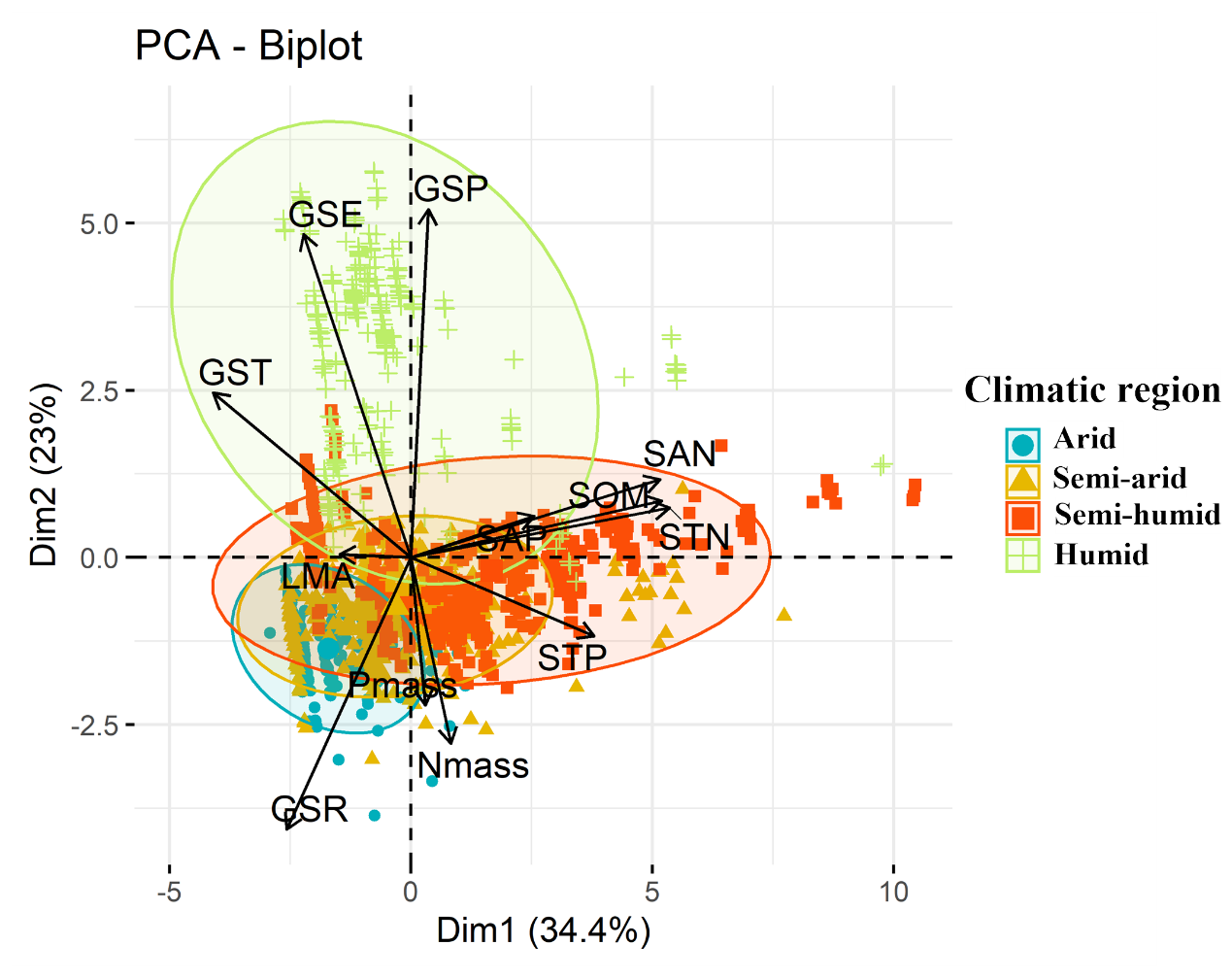


**Supplementary Figure 2.** Principal component analyses (PCA) with all potential explanatory variables pooled together in four China’s climatic regions. LMA, leaf mass per area; N_mass_, mass-based leaf nitrogen; P_mass_, mass-based leaf phosphorus; SOM, soil organic matter; STN, soil total nitrogen; STP, soil total phosphorus; SAN, soil available nitrogen; SAP, soil available phosphorus; GST, growing season temperature; GSP, growing season precipitation; GSE, growing season evapotranspiration; GSR, growing season solar radiation.


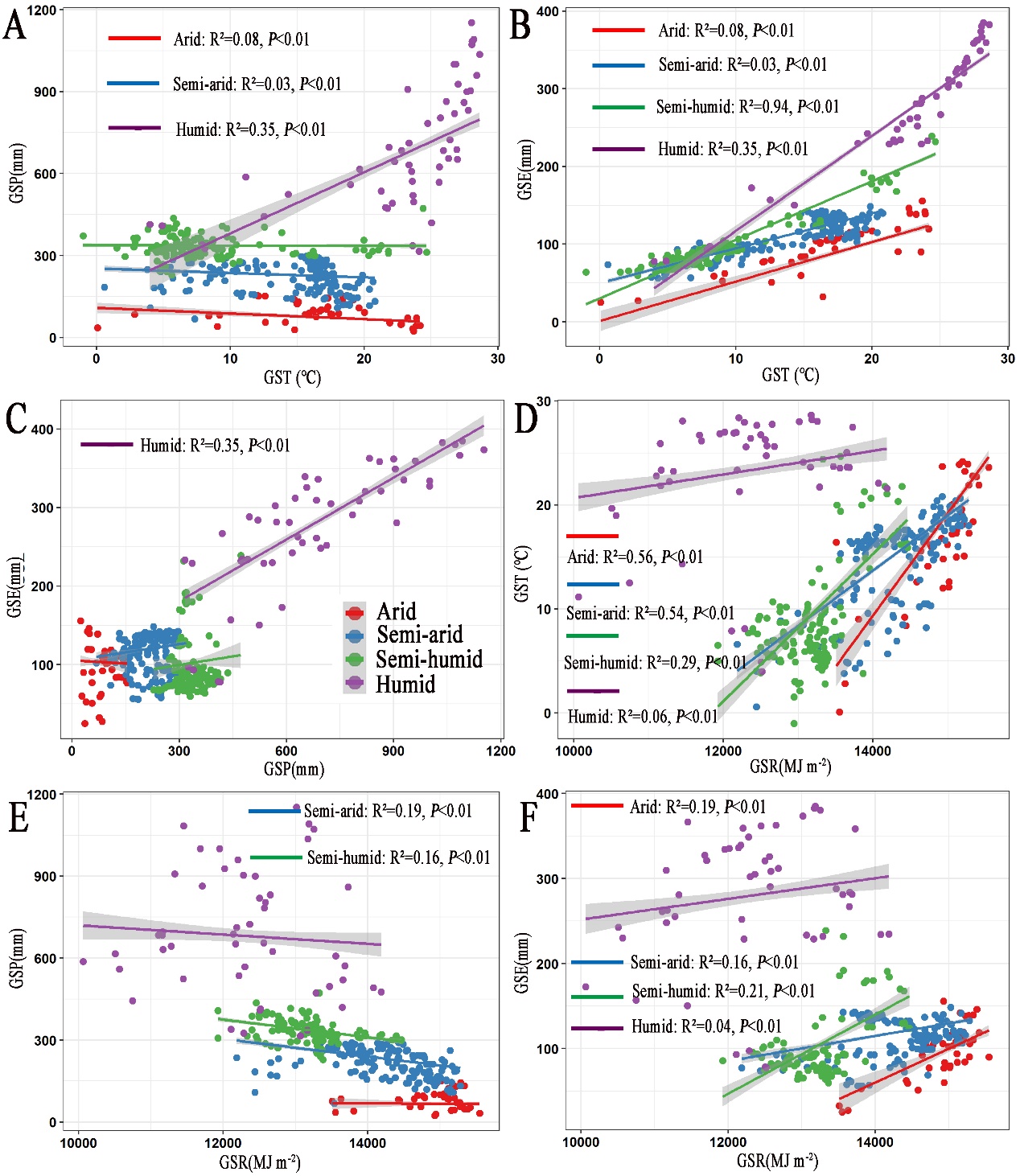
 **Supplementary Figure 3.** Relationships of climatic variables in four climatic regions across China. GST, growing season temperature; GSP, growing season precipitation; GSE, growing season evapotranspiration; GSR, growing season solar radiation.


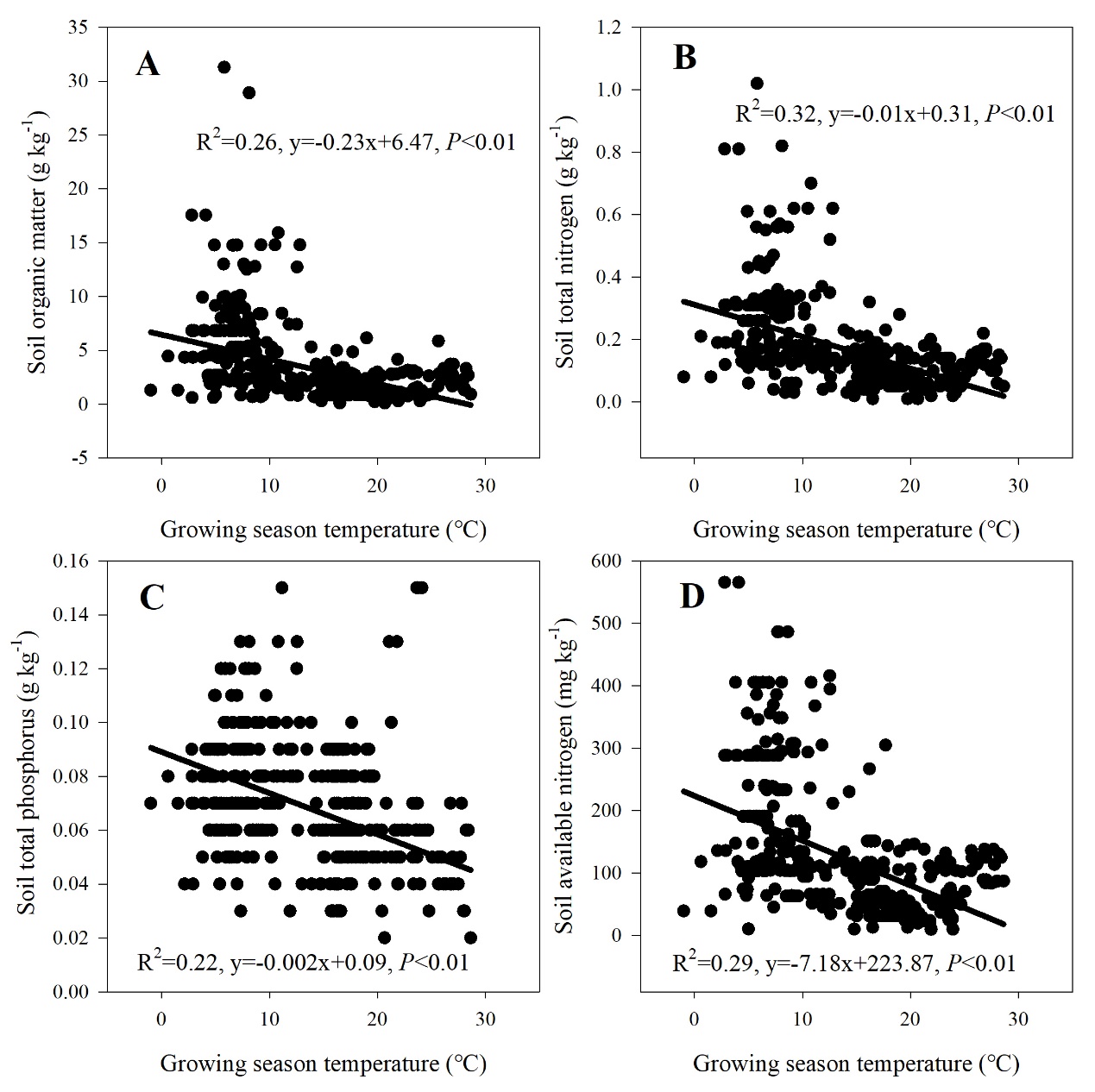


**Supplementary Figure 4.** Relationships of growing season temperature with soil nutrients (A, soil organic matter; B, soil total nitrogen; C, soil total phosphorus; D, soil available nitrogen) in our sampling sites across China.


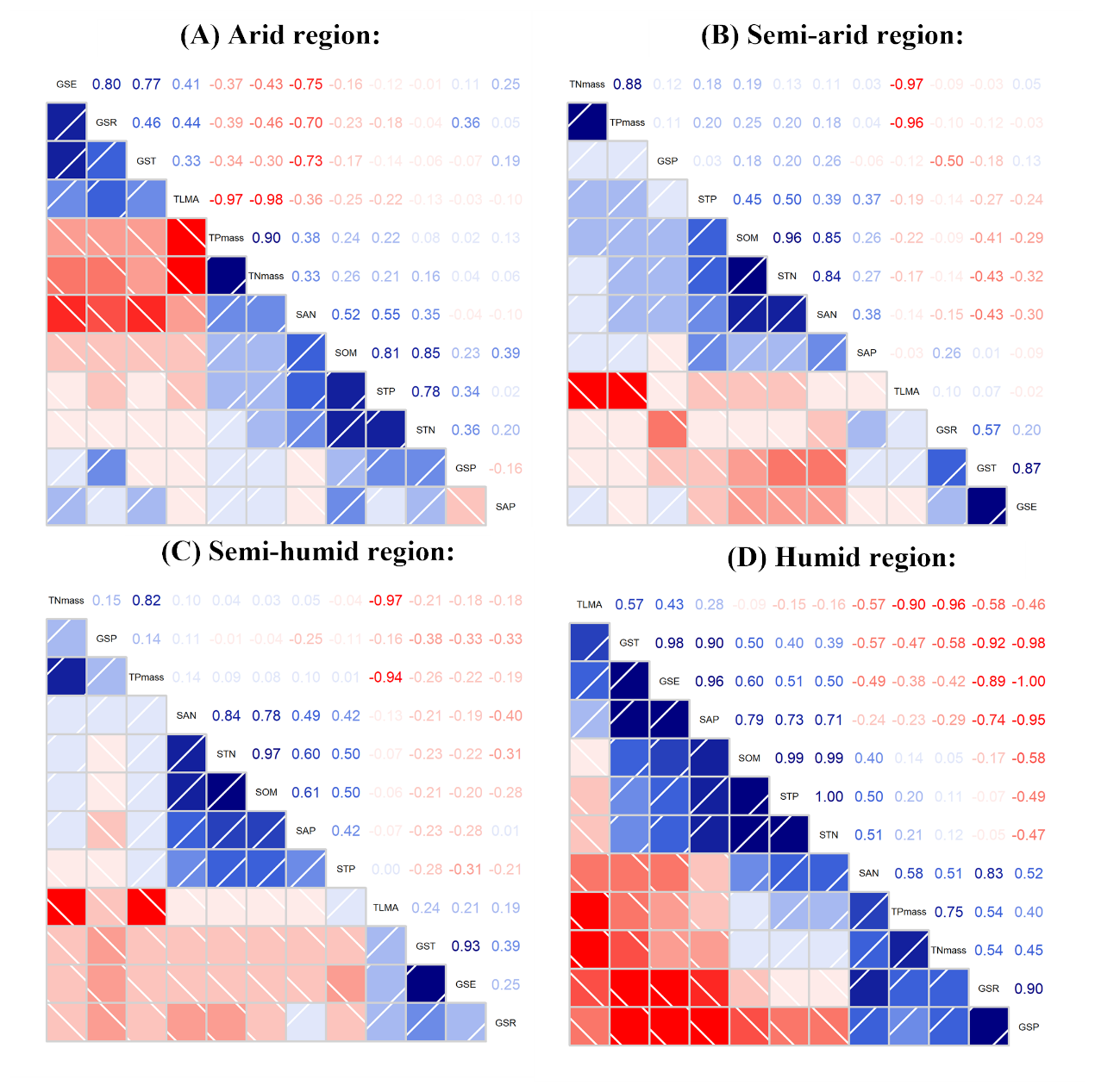


**Supplementary Figure 5.** Correlogram of intercorrelations among relative benefits of leaf traits, soil properties, and climate factors in four China’s climatic regions. The blue and red colors indicate positive and negative relationships, respectively. The color depth represents correlated strength, that is, a deep color suggests a strong correlation. TLMA, TN_mass_, and TP_mass_ are relative benefits of leaf mass per area; mass-based leaf nitrogen and mass-based leaf phosphorus, respectively; SOM, soil organic matter; STN, soil total nitrogen; STP, soil total phosphorus; SAN, soil available nitrogen; SAP, soil available phosphorus; GST, growing season temperature; GSP, growing season precipitation; GSE, growing season evapotranspiration; GSR, growing season solar radiation.

**
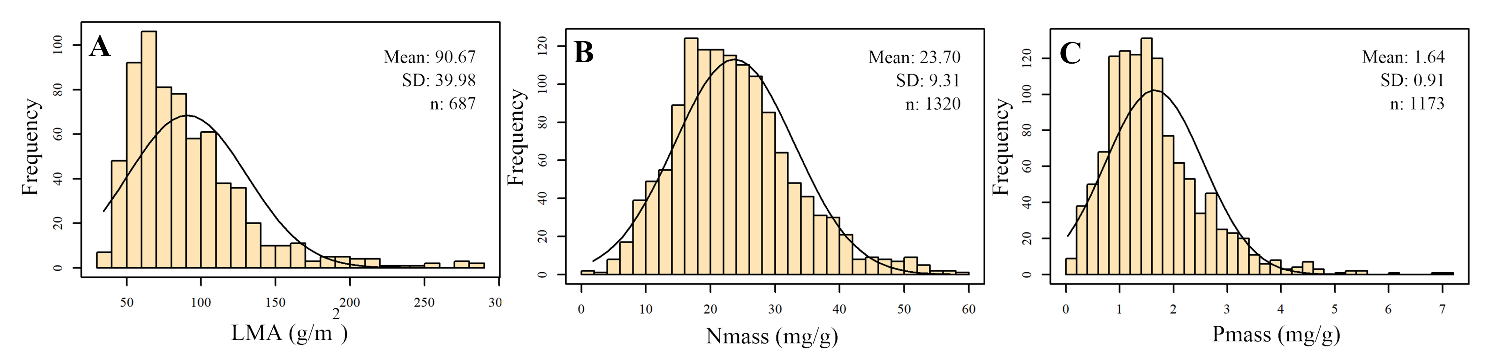
**

**Supplementary Figure 6.** Frequency distribution of herbaceous plant LMA, N_mass_, P_mass_ among four China’s climatic regions


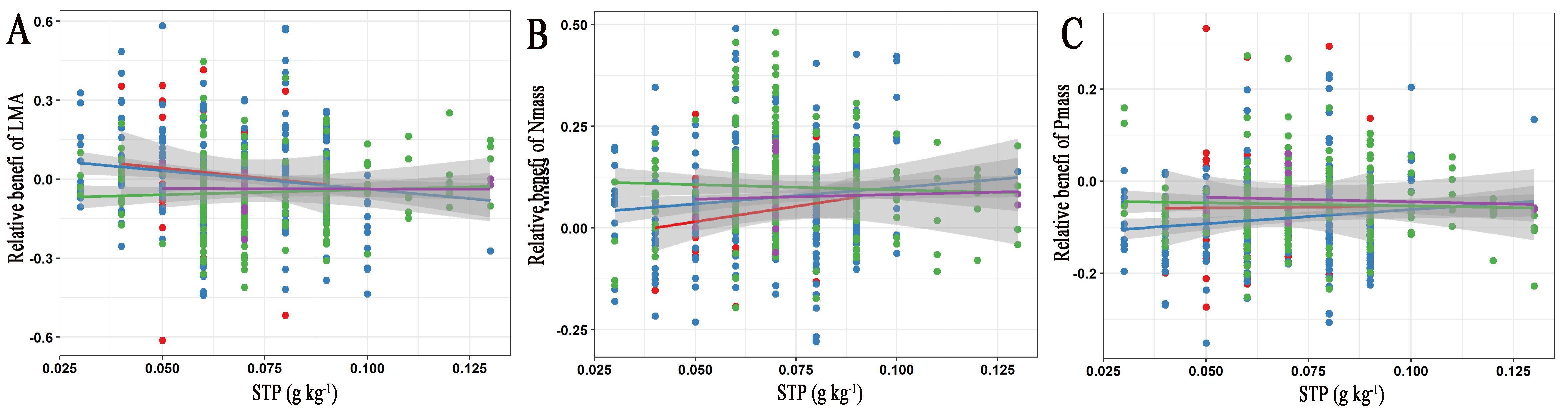


**Supplementary Figure 7.** Relationships between the relative benefits of leaf traits and soil total phosphorus. The red, blue, green, and purple colours represent the arid, semi-arid, semi-humid, and humid climatic region, respectively. LMA, leaf mass per area; N_mass_, mass-based leaf nitrogen; P_mass_, mass-based leaf phosphorus; STP, soil total phosphorus. The statistical information of linear regression are shown in Table 3.

## Supplementary Tables

**Supplementary Table 1.** The mean values and results of the analysis of variance (ANOVAs) for soil properties and climatic factors in four China’s climatic regions

| **Climatic regions** | **SOM (g kg^-1^)** | **STN**  **(g kg^-1^)** | **STP**  **(g kg^-1^)** | **SAN**  **(mg kg^-1^)** | **SAP**  **(mg kg^-1^)** | **GST (℃)** | **GSP (mm)** | **GSE (mm)** | **GSR**  **(MJ m^-2^)** |
| --- | --- | --- | --- | --- | --- | --- | --- | --- | --- |
| Arid region | 0.66a | 0.05a | 0.05a | 31.05a | 5.28a | 20.16a | 66.71a | 103.55a | 15094.83a |
| Semi-arid region | 2.22b | 0.13b | 0.07b | 87.60b | 4.89b | 15.85b | 226.54b | 120.92b | 14402.80b |
| Semi-humid region | 4.77c | 0.23c | 0.08c | 173.65c | 5.09ab | 9.55c | 336.43c | 102.26a | 13189.30c |
| Humid region | 2.83d | 0.14b | 0.05a | 117.86d | 5.60c | 23.35d | 680.20d | 280.59c | 12360.17d |

Abbreviations: SOM, soil organic matter; STN, soil total nitrogen; STP, soil total phosphorus; SAN, soil available nitrogen; SAP, soil available phosphorus; GST, growing season temperature; GSP, growing season precipitation; GSE, growing season evapotranspiration; GSR, growing season solar radiation. Differences among the four climatic regions are shown with different letters according to the least significant difference (LSD) tests (*P*<0.05).
