# Supplementary material for: Context-Dependency in Relationships Between Herbaceous Plant Leaf Traits and Abiotic Factors
Source: Front Plant Sci. 2022 Mar 25;13:757077. doi: 10.3389/fpls.2022.757077 (PMC8990845; doi:10.3389/fpls.2022.757077)
Supplement: Supplementary file 2 [file Data_Sheet_2.docx]

Supplementary Material 2

A list of papers from which the data were extracted for this meta-analysis.

Baumann, F., He, J. S., Schmidt, K., Kühn, P., and Scholten, T. (2009). Pedogenesis, permafrost, and soil moisture as controlling factors for soil nitrogen and carbon contents across the Tibetan Plateau. Global Change Biology 15, 3001-3017.

Baumann, F., Schmidt, K., Dörfer, C., He, J. S., Scholten, T., and Kühn, P. (2014). Pedogenesis, permafrost, substrate and topography: plot and landscape scale interrelations of weathering processes on the central-eastern Tibetan Plateau. Geoderma 226, 300-316.

Dörfer, C., Kühn, P., Baumann, F., He, J. S., and Scholten, T. (2013). Soil organic carbon pools and stocks in permafrost-affected soils on the Tibetan Plateau. Plos One 8, e57024.

Geng, Y., Baumann, F., Song, C., Zhang, M., Shi, Y., Kühn, P., Scholten, T., and He, J. S. (2017). Increasing temperature reduces the coupling between available nitrogen and phosphorus in soils of Chinese grasslands. Scientific Reports 7, 43524.

Geng, Y., Wang L., Jin, H., Liu, Y., and He J. S. (2014). Alpine climate alters the relationships between leaf and root morphological traits but not chemical traits. Oecologia 175, 445-455.

Geng, Y., Wang, Z. H., Liang, C. Z., Fang, J. Y., Baumann, F., Kühn, P., Scholten, T., and He, J. S. (2012). Effect of geographical range size on plant functional traits and the relationships between plant, soil and climate in Chinese grasslands. Global Ecology and Biogeography 21, 416-427.

Geng, Y., Ma, W. H., Wang, L., Baumann, F., Kühn, P., Scholten, T., and He, J. S. (2017). Linking above- and belowground traits to soil and climate variables: an integrated database on China's grassland species. Ecology 98, 1471.

Han, W. X., Fang, J. Y., Guo, D. and Zhang, Y. (2005). Leaf nitrogen and phosphorus stoichiometry across 753 terrestrial plant species in China. New Phytologist 168(2), 377-385.

He, J. S., Fang, J. Y., Wang, Z. H., Guo, D. L., Flynn, D. F. B., and Geng, Z. (2006a). Stoichiometry and large-scale patterns of leaf carbon and nitrogen in the grassland biomes of China. Oecologia 149, 115-122.

He, J. S., Wang, L., Flynn, D. F. B., Wang, X. P., Ma, W. H., and Fang, J. Y. (2008). Leaf nitrogen: phosphorus stoichiometry across Chinese grassland biomes. Oecologia 155, 301-310.

He, J. S., Wang, X. P., Flynn, D. F. B., Wang, L., Schmid, B., and Fang, J. Y. (2009). Taxonomic, phylogenetic, and environmental trade-offs between leaf productivity and persistence. Ecology 90, 2779-2791.

He, J. S., Wang, X. P., Schmid, B., Flynn, D. F. B., Li, X. F., Reich, P. B., and Fang, J. Y. (2010). Taxonomic identity, phylogeny, climate and soil fertility as drivers of leaf traits across Chinese grassland biomes. Journal of Plant Research 123, 551-561.

He, J. S., Wang, Z. H., Wang, X. P., Schmid, B., Zuo, W. Y., Zhou, M., Zheng, C. Y., Wang, M. F., and Fang, J. Y. (2006b). A test of the generality of leaf trait relationships on the Tibetan Plateau. New Phytologist 170, 835-848.

Shi, Y., Baumann, F., Ma, Y., Song, C., Kühn, P., Scholten, T., and He, J. S. (2012). Organic and inorganic carbon in the topsoil of the Mongolian and Tibetan grasslands: pattern, control and implications. Biogeosciences 9, 2287-2299.

Shi, Y, Wang, Y. H., Ma, Y. L., Ma, W. H., Liang, C. Z., Flynn, D. F. B., Schmid, B., Fang, J. Y., and He, J. S. (2014). Field-based observations of regional-scale, temporal variation in net primary production in Tibetan alpine grasslands. Biogeosciences 11, 2003-2016.

Yang, X. X, Yang, Y., Ji, C. J., Feng, T., Shi, Y., Lin, L., Ma, J. J., and He, J. S. (2014). Large-scale patterns of stomatal traits in Tibetan and Mongolian grassland species. Basic and Applied Ecology 15, 122-132.
